# Remote Monitoring by ViQtor Upon Implementation on a Surgical Department (REQUEST-Trial): Protocol for a Prospective Implementation Study

**DOI:** 10.2196/70707

**Published:** 2025-07-03

**Authors:** Ephrahim E Jerry, Arthur R Bouwman, Simon W Nienhuijs

**Affiliations:** 1 Department of Surgery Catharina Ziekenhuis Eindhoven The Netherlands; 2 Department of Anestesiology Catharina Ziekenhuis Eindhoven The Netherlands

**Keywords:** monitoring, vital signs, early warning system, implementation, wearable device, workload

## Abstract

**Background:**

Continuous monitoring of patients’ vital signs is critical for early detection of postoperative complications. Traditional manual monitoring by nursing staff is time-consuming and provides only intermittent data. Wearable devices offer continuous monitoring capabilities, potentially enhancing early warning systems, reducing nurse workload, and facilitating earlier patient discharge. However, research on their implementation and effectiveness in clinical settings remains limited.

**Objective:**

This study aims to evaluate the implementation and feasibility of continuous monitoring using photoplethysmography sensor technology (a viQtor device) in a surgical ward. We will also assess the impact on nursing workload and the usability of this workflow.

**Methods:**

The REQUEST (Remote Monitoring by viQtor Upon Implementation on a Surgical Department) study is a prospective observational implementation study conducted over 8 months in a surgical ward. The vital signs of 500 postoperative patients will be continuously monitored using the viQtor device, which measures heart rate, respiratory rate, and oxygen saturation. The study consists of 2 phases: an initial period with manual spot checks, followed by a phase using the wearable device as the primary monitoring tool. Outcomes include the Integrated Workload Scale for nursing workload and a framework evaluating acceptability, feasibility, adoption, and sustainability. Data collection involves device performance metrics, questionnaires (the Evidence-Based Practice Attitude Scale and the System Usability Scale), and thematic analysis of focus groups.

**Results:**

Staff training was completed in October 2024, and full implementation is ongoing. Preliminary findings, including data on usability and workload, are expected by July 2025.

**Conclusions:**

This study will provide insight into the practical implementation of continuous vital sign monitoring in surgical care. The findings may support future adoption of wearable technology in clinical workflows.

**Trial Registration:**

ClinicalTrials.gov NCT06574867; https://clinicaltrials.gov/study/NCT06574867

**International Registered Report Identifier (IRRID):**

DERR1-10.2196/70707

## Introduction

The health care sector is currently undergoing a significant transformation, with increasing emphasis on integrating smart technologies to enhance patient safety and clinical efficiency. A central innovation in this shift is the use of wearable monitoring devices in hospitals, particularly for patients recovering from major abdominal surgery. These procedures are associated with a high risk of serious adverse events, which could lead to preventable harm if early signs of clinical deterioration—often evident in vital sign changes—go unnoticed [1].

Current early warning systems are based on intermittent manual assessments of vital signs, and the Early Warning Score (EWS), typically performed three times daily. However, research indicates that deterioration in vital parameters often precedes clinical events by several hours. One study found that in approximately 60% of patients who experienced postoperative complications, changes in vital signs had already occurred but were not recognized in time due to the limited frequency of monitoring [2].

Efforts have been made to improve the EWS, such as the development of the Modified Early Warning Score (MEWS), which incorporates oxygen saturation [3,4]. While these adaptations have increased the sophistication of monitoring, the overall predictive value of these systems remains limited [2,5]. Moreover, variation in EWS methodologies and inconsistent validation have raised concerns about reliability in daily clinical practice [6].

Advancements in wearable technology offer a promising alternative. These devices provide real-time, continuous monitoring of vital signs, enabling earlier detection of clinical deterioration and potentially more timely intervention [4,7-9]. In addition, continuous monitoring may reduce the burden of manual assessments on nursing staff. Despite the potential benefits, widespread implementation of wearables in clinical settings remains uncommon. Most studies to date have focused on technical performance, while few have explored how such devices are adopted and integrated into routine care.

Successfully introducing wearable monitoring systems requires attention not only to the technology itself but also to the clinical environment, the needs and attitudes of health care professionals, and the sustainability of new workflows. This study therefore follows the revised Medical Research Council framework for evaluating complex interventions [10], which emphasizes the role of context, stakeholder engagement, and real-world feasibility.

The objective of this study is to evaluate the implementation and feasibility of continuous vital sign monitoring using the viQtor wearable device in a surgical ward. In doing so, the study aims to assess the device’s usability, its impact on nursing workload, and how effectively it can be embedded in standard clinical practice.

## Methods

### Setting

This prospective implementation study with retrospective data analysis (the REQUEST [Remote Monitoring by viQtor Upon Implementation on a Surgical Department] trial) will be conducted at a large teaching hospital. All adult patients (aged 18 years and older) admitted to the surgical department for elective procedures will be eligible for inclusion, provided they meet our inclusion criteria and provide informed consent. Patients unable to wear the device or unwilling to participate will be excluded.

**Figure 1:**
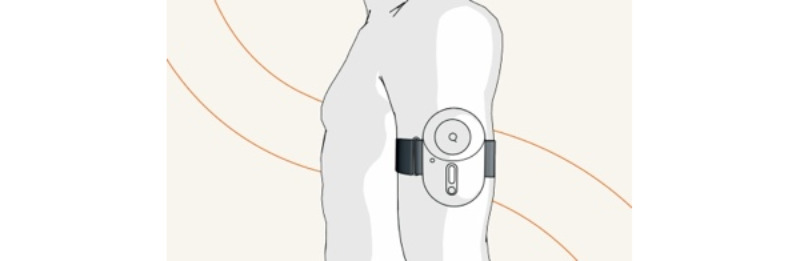
The viQtor device placed on the upper arm.

The viQtor device, developed by SMARTCARE, was selected due to its proven capability for autonomous, continuous monitoring and its established structure for seamless data transfer into the electronic health record (EHR). The device is CE (Conformité Européenne)-certified and transmits data every minute via a cellular network without the need for any hub. Each vital sign—heart rate, respiratory rate, and peripheral oxygen saturation (SpO₂)—is updated every minute, with data being transferred to the EHR. The device provides median values from the last 4 hours for each vital sign, which means that the system aggregates data over that period to ensure stability in measurements. In the event of missing data, the system generates flags to indicate the absence of readings, and data imputation strategies will be applied. By using clinical-grade photoplethysmography sensor technology, the device allows accurate tracking of heart rate, respiratory rate, and SpO₂. Although the device can also measure skin temperature, this parameter will not be used in this study. The solution comprises a device with a charger and an armband. The device is worn on the upper arm under clothing and is applied upon admission (Figure 1).

### Implementation Phase

Implementation of the device as the new standard of care for monitoring will be executed in 2 distinct phases to ensure a thorough evaluation and implementation of this monitoring system (Table 1).

During phase 1 (the initial phase; months 1-3), patients will continue to receive the current standard of care, which involves manual spot checks and MEWS measurements by nursing staff. Concurrently, all health care staff will receive training on the application, data interpretation, and protocol adherence concerning the solution to optimize the infrastructure before its implementation.

Following phase 1 (in month 4; the evaluation and validation period), the preimplementation phase will be evaluated. Predictive accuracy will be evaluated by comparing device data against clinical outcomes such as complications, intensive care unit transfers, or MEWS-triggered interventions using area under the receiver operating characteristic curve analysis.

In phase 2 (months 5-8), the wearable will become the primary tool for patient monitoring, supplemented by manual spot checks by nurses as needed. This phase aims to evaluate the integration into standard care.

During the whole study, every minute data are securely stored. During phase 2 of the study, 6 times a day, median values of the previous 4-hour window will be uploaded to the EHR. In case of signal loss or low-quality readings, the system generates flags, allowing for assessment of signal-to-noise ratio (SNR).

**Table 1:** Psychometric properties and statistical analysis.

Objective	Instrument	Variable	Statistical test	Achievement criteria
Fidelity	SUS^a^	Continuous	Unpaired *t* test or Mann-Whitney *U* test	Fidelity achieved when protocol adherence is ≥95%
Acceptance	SC^b^/day	Continuous	Descriptive statistics	Acceptance achieved when ≥75% of nurses use the device regularly
Acceptance	SC/patient	Continuous	Descriptive statistics	Acceptance achieved when ≥75% of nurses use the device regularly
Acceptance	IWS^c^	Ordinal	1-way ANOVA	Acceptance achieved when ≥75% of nurses use the device regularly
Adoption	EBPAS^d^	Continuous	Repeated measures ANOVA	Adoption achieved when device use exceeds 70% by clinical staff
Appropriateness	SNR^e^	Continuous	Descriptive statistics	Adoption achieved when device use exceeds 70% by clinical staff
Appropriateness	ADE^f^	Continuous	Descriptive statistics	Appropriateness achieved with minimal ADEs
Feasibility	Thematic analysis	Nominal	Braun and Clarke [11] thematic analysis	Feasibility achieved when 80% of clinicians can successfully implement the device
Time efficiency	Time measurement	Continuous	Descriptive statistics	Time efficiency achieved when nurses’ time spent on monitoring decreases by ≥20%
Predictive accuracy	MEWS^g^ and CREWS^h^	Nominal	AUROC^i^	Predictive accuracy achieved when AUROC ≥0.80

^a^SUS: System Usability Scale.

^b^SC: spot check.

^c^IWS: Integrated Workload Scale.

^d^EBPAS: Evidence Based Practice Attitude Scale.

^e^SNR: signal-to-noise ratio.

^f^ADE: adverse device event.

^g^MEWS: Modified Early Warning Score.

^h^CREWS: Continuous Remote Early Warning Score.

^i^AUROC: area under the receiver operating characteristic curve.

### Outcomes

The primary objective of this study is to evaluate the implementation of a new wearable on the surgical ward at Catharina Hospital Eindhoven. This will be achieved by assessing its usability, degree of implementation, and the workload for nursing staff. The primary outcome will be acceptability, specifically evaluating workload as measured by the Integrated Workload Scale (IWS). The IWS is a comprehensive tool that provides multidimensional descriptions and gradations of workload, ranging from “not demanding” to “work too demanding” on a 9-point Likert scale. This scale is suitable for both periodic assessment and real-time monitoring of tasks, with measurements conducted throughout the entire study period.

For secondary outcomes, the study will apply the framework of the taxonomy of implementation outcomes of Proctor et al [12], focusing on adoption, feasibility, appropriateness, predictive accuracy, and technical feasibility. Adoption will be measured by the Evidence-Based Practice Attitude Scale and the System Usability Scale, along with the amount of spot checks conducted in addition to the standard monitoring.

Feasibility will be evaluated by determining the extent to which health care employees can successfully implement the device, and by measuring the time required to perform and process manual spot checks or digital monitoring checks. Appropriateness will be assessed by examining the SNR and counting the number of (suspected) adverse events that occur during or result from the use of the wearable.

Additionally, the predictive accuracy for early detection of health deterioration and postoperative complications among patients will be evaluated through focus group discussions. Technical feasibility will involve analyzing the availability of data on the web platform, including monitoring time and the types and quantities of algorithm artifacts, such as low-quality data during the monitoring of photoplethysmography values.

Approximately 70 nurses from the surgical ward will participate in the study and complete the IWS. In addition, 10 nurses and 5 physicians will voluntarily join focus groups to conduct an in-depth evaluation of the new implementation. These focus groups, which will be established prior to the study’s commencement, will meet 3 times during the study period to discuss the progress of the study and share their experiences with the implementation. A structured analysis of fidelity, feasibility, and acceptability will be conducted by the focus group, and this information will be processed and objectified using a thematic analysis devised by Braun and Clarke [11].

### Statistical Analysis

Quantitative data will be analyzed using IBM SPSS Statistics (version 26.0). Descriptive statistics will be used to summarize categorical data (frequencies and percentages) and continuous variables (means and SD or medians and IQRs, depending on the distribution). Normality will be assessed using the Shapiro-Wilk test. Group comparisons will be made using *t* tests, ANOVA, Mann-Whitney *U*, or chi-square tests where appropriate. For repeated measures, linear mixed models or repeated measures ANOVA will be used. Qualitative data from focus groups will be coded and thematically analyzed following Braun and Clarke’s [11] approach.

### Ethical Considerations

This study involves human participants and has been approved by Medical Research Ethics Committees United (reference number W24.065). Written informed consent to participate will be obtained from all participants prior to inclusion in the study.

All data collected will be pseudonymized before analysis to protect participants’ privacy. Personal identifiers will be replaced with unique study codes, and the key linking codes to participant identities will be stored separately in a secure, access-restricted location. Only authorized study personnel will have access to this information.

Participants will not receive any financial compensation or other incentives for participation in this study. Participation is voluntary, and participants may withdraw at any time without any consequences for their care.

## Results

Staff training was completed in October 2024, and full implementation is ongoing. Up to 150 participants intend to be enrolled in the study. Preliminary findings, including data on usability and workload, are expected by July 2025 (see details in Table 2).

**Table 2:** Measurement timetable for psychometric properties.

Objective	Instrument	0^a^	1	2	3	4 (evaluation/validation)	5	6	7	8 (evaluation/validation)
Fidelity	SUS^b^						Q^c^			Q
Acceptance	SC^d^/day				AD^e^					AD
Acceptance	SC/patient				AD					AD
Acceptance	IWS^f^	Q			Q		Q	Q	Q	Q
Adoption	EBPAS^g^	Q			Q				Q	Q
Appropriateness	SNR^h^									AD
Appropriateness	ADE^i^									AD
Feasibility	Thematic analysis				FG^j^			FG		FG
Time efficiency	Time measurement		M^k^	M	M		M	M	M	M
Predictive accuracy	MEWS^l^ and CREWS^m^		M	M	M	AD and M	AD (and M)	AD (and M)	AD	AD

^a^Numbers 0-8 represent the study months, starting from baseline (month 0) through to month 8.

^b^SUS: System Usability Scale.

^c^Q: questionnaire.

^d^SC: spot check.

^e^AD: administered data.

^f^IWS: Integrated Workload Scale.

^g^EBPAS: Evidence Based Practice Attitude Scale.

^h^SNR: signal-to-noise ratio.

^i^ADE: adverse device event.

^j^FG: focus group.

^k^M: manual measurement.

^l^MEWS: Modified Early Warning Score.

^m^CREWS: Continuous Remote Early Warning Score.

## Discussion

This study is expected to demonstrate that the implementation of continuous vital sign monitoring using the viQtor wearable device is feasible within a surgical ward setting. We anticipate that this technology will be perceived as acceptable and usable by health care professionals, and that its integration may contribute to a measurable reduction in perceived nursing workload. Importantly, by including continuous SpO₂ monitoring, the system may offer added clinical value in identifying early signs of postoperative deterioration.

While earlier studies have assessed the technical accuracy of wearable monitoring devices, relatively few have focused on their integration into clinical workflows. For instance, Breteler et al [8] validated a similar system in high-risk surgical patients, but implementation outcomes such as usability or staff adoption were not the focus. Bellomo et al [7] also examined automated advisory systems, but their routine use in general hospital wards remains uncommon.

Our study distinguishes itself by applying a structured implementation framework, specifically the revised Medical Research Council guidance [10] and the taxonomy of implementation outcomes of Proctor et al [12]. This approach allows us to assess whether the device not only works but also can be realistically adopted in daily practice.

A notable addition in our protocol is the continuous monitoring of SpO₂, a parameter that was lacking in earlier studies such as those involving a Healthdot device. Since respiratory complications are among the more frequent and severe postoperative issues, continuous SpO₂ tracking may enhance the early recognition of patient decline [4,6,13].

The study’s multidisciplinary design, combining clinical, technical, and implementation expertise could be seen as a strength. The use of validated instruments—including the IWS [14], Evidence-Based Practice Attitude Scale [12], and the System Usability Scale—ensures a structured and multidimensional evaluation.

Limitations of this study can be expected as well. As a single-center trial focusing on patients undergoing elective surgery, its findings may not be directly generalizable to other hospital departments or to emergency or critical care populations. Additionally, while the viQtor device captures data at regular intervals, data loss or reduced signal quality could occur. These technical aspects will be explored through SNR analysis and artifact tracking. If implementation proves successful, the findings may support broader deployment across other wards or institutions. Future research could explore postdischarge remote monitoring or the integration of wearable data into clinical decision support systems, possibly enhanced by predictive algorithms.

Results will be shared internally within the hospital through clinical meetings and departmental presentations. Externally, we plan to present findings at national conferences and submit our results to peer-reviewed journals. Data will be made available upon reasonable request, and the implementation protocol may serve as a blueprint for similar projects in other hospitals.

## Abbreviations

CE: Conformité Européenne
EHR: electronic health record
EWS: Early Warning Score
IWS: Integrated Workload Scale
MEWS: Modified Early Warning Score
REQUEST: Remote Monitoring by viQtor Upon Implementation on a Surgical Department
SNR: signal-to-noise ratio
SpO₂: peripheral oxygen saturation

## Data Availability

The datasets used and/or analyzed during this study are available from the corresponding author on reasonable request. All data generated or analyzed during this study are included in this published article.

Feasibility will be assessed based on the proportion of eligible patients enrolled, device adherence rates, and completeness of data collection. Acceptability will be evaluated through surveys and interviews with health care professionals and patients regarding their experiences with the new device.

## References

[1] Chen L, Zheng H, Chen L, Wu S, Wang S (2021). National Early Warning Score in predicting severe adverse outcomes of emergency medicine patients: A retrospective cohort study. JMDH.

[2] Majumder S, Mondal T, Deen M (2017). Wearable sensors for remote health monitoring. Sensors (Basel).

[3] Smith GB, Prytherch DR, Meredith P, Schmidt PE, Featherstone PI (2013). The ability of the National Early Warning Score (NEWS) to discriminate patients at risk of early cardiac arrest, unanticipated intensive care unit admission, and death. Resuscitation.

[4] Jacobs F, Scheerhoorn J, Mestrom E, van der Stam J, Bouwman RA, Nienhuijs S (2021). Reliability of heart rate and respiration rate measurements with a wireless accelerometer in postbariatric recovery. PLoS One.

[5] Patel V, Orchanian-Cheff A, Wu R (2021). Evaluating the validity and utility of wearable technology for continuously monitoring patients in a hospital setting: Systematic review. JMIR Mhealth Uhealth.

[6] Gerry S, Bonnici T, Birks J, Kirtley S, Virdee PS, Watkinson PJ, Collins GS (2020). Early warning scores for detecting deterioration in adult hospital patients: systematic review and critical appraisal of methodology. BMJ.

[7] Bellomo R, Ackerman M, Bailey M, Beale R, Clancy G, Danesh V, Hvarfner A, Jimenez E, Konrad D, Lecardo M, Pattee KS, Ritchie J, Sherman K, Tangkau P (2012). A controlled trial of electronic automated advisory vital signs monitoring in general hospital wards*. Crit Care Med.

[8] Breteler M, KleinJan E, Dohmen D, Leenen L, van Hillegersberg Richard, Ruurda J, van Loon Kim, Blokhuis Taco J, Kalkman Cor J (2020). Vital signs monitoring with wearable sensors in high-risk surgical patients: A clinical validation study. Anesthesiology.

[9] Weenk M, Koeneman M, van de Belt TH, Engelen LJ, van Goor H, Bredie SJ (2019). Wireless and continuous monitoring of vital signs in patients at the general ward. Resuscitation.

[10] Manetti S, Vainieri M, Guidotti E, Zuccarino S, Ferré Francesca, Morelli MS, Emdin M (2020). Research protocol for the validation of a new portable technology for real-time continuous monitoring of Early Warning Score (EWS) in hospital practice and for an early-stage multistakeholder assessment. BMJ Open.

[11] Braun V, Clarke V (2006). Using thematic analysis in psychology. Qual Res Psychol.

[12] Proctor E, Silmere Hiie, Raghavan Ramesh, Hovmand Peter, Aarons Greg, Bunger Alicia, Griffey Richard, Hensley Melissa (2011). Outcomes for implementation research: conceptual distinctions, measurement challenges, and research agenda. Adm Policy Ment Health.

[13] Taenzer A, Pyke J, McGrath S, Blike G (2010). Impact of pulse oximetry surveillance on rescue events and intensive care unit transfers. Anesthesiology.

[14] Kramer R, Johnson A, Zeilstra M (2015). How Busy is Too Busy? Validation of the Dutch Integrated Workload Scale. Proceedings of Fifth International Rail Human Factors Conference.

